# Population dynamics of the Teak defoliator (*Hyblaea puera *Cramer) in Nilambur teak plantations using Randomly Amplified Gene Encoding Primers (RAGEP)

**DOI:** 10.1186/1472-6785-5-1

**Published:** 2005-02-02

**Authors:** N Chandrasekhar, TV Sajeev, VV Sudheendrakumar, Moinak Banerjee

**Affiliations:** 1 Human Molecular Genetics Lab, Rajiv Gandhi Centre for Biotechnology, Poojapura, Trivandrum, Kerala- 695012, India; 2 Division of Entomology, Kerala Forest Research Institute, Peechi, Kerala- 680653, India

## Abstract

**Background:**

The Teak defoliator (*Hyblaea puera*) is a pest moth of teak woodlands in India and other tropical regions (e.g. Thailand) and is of major economic significance. This pest is of major concern as it is involved in complete defoliation of trees during the early part of the growing season. Defoliation does not kill teak trees, but it results in huge amount of timber loss. Teak defoliator outbreaks are a regular annual feature in most teak plantations in India and it is extremely difficult to predict the exact time and place of occurrence of these outbreaks. Evidence from the study of the population dynamics of *H. puera *indicated habitual, short range movements of emerging moth populations, suggesting that these populations have spread to larger areas, generation after generation, affecting the entire teak plantations. We were therefore interested in investigating the temporal and spatial relationship among various population groups in Nilambur, Kerala (India) and address the cause of outbreak at the landscape level.

**Results:**

The populations were classified into 'endemic', 'epicenter' and 'epidemic' populations based on the time of occurrence and size of infestation. We devised a novel method of screening nuclear and mitochondrial DNA polymorphisms using Randomly Amplified Gene Encoding Primers (RAGEP). We have used this method extensively to evaluate the species specificity, reproducibility and to discriminate among the three different characterised populations of teak defoliator.

**Conclusions:**

This method also allowed us to comment with some certainty that the endemic teak defoliator, *H. puera *do not play a major role in contributing to large-scale infestations. With respect to the hypotheses put forward regarding the origin of outbreaks of the moth, this study confirms the role of migration in outbreak causation, while negating the belief that endemic populations aggregate to cause an epidemic.

## Background

Teak (*Tectona grandis *L.) is a very valuable timber species, and is a member of the moist deciduous and dry deciduous forest types. Teak plantations are threatened by two major pests: the Teak Defoliator (*Hyblaea puera *Cramer) Lepidoptera: Hyblaeidae and the Teak Skeletonizer (*Eutectona machaeralis *(Walker) syn.). *H. puera *is less widely distributed in the tropics: in Oriental and Australian regions (India, Sri Lanka, Burma, Java, Papua New-Guinea, Northern Queensland in Australia, Solomon Islands); in the West Indies; and in South and parts of East Africa [1]. The Teak defoliator is of major concern since it is involved in complete defoliation of trees during the early part of the growing season. Defoliation does not kill the trees, but does lead to huge timber loss. Recent studies have shown that the defoliation leads to an average loss of 44% of the potential volume increment in four to nine year-old teak plantations. It has been estimated that in the Nilambur teak plantation during the study period, protected trees increased by an annual increment of 6.7 m^3^/ha compared with 3.7 m^3^/ha for unprotected trees, a gain of 3 m^3^/ha per annum [2].

Teak defoliator outbreaks are a regular annual feature in teak plantations in Kerala, India. It is difficult to predict the exact time and place of these outbreaks. Evidence gathered from the past decade on the population dynamics of *H. puera *indicates habitual, short range movements of emerging moth populations, suggesting that these spread to larger areas, generation after generation, affecting entire teak plantations [3]. Earlier studies also indicated that the outbreaks begin as small epicenters during the pre-monsoon season [4]. Populations were classified as 'endemic', 'epicenter' and 'epidemic', based on their time of occurrence and the density of the population as represented by the area it infests. Endemics are insects belonging to the low-density population level; epicenters are patchy, medium density outbreaks that occur during the pre-monsoon season, whilst epidemic represents large area, high-density outbreak populations. An understanding of the origin and spread of the epidemic of this pest, which erupt suddenly following the pre-monsoon rain each year, is an important prerequisite for developing appropriate control strategies. If progenies of the epicenter populations cause the larger epidemics, control of these could prevent major outbreaks. On the other hand, if immigrant moths were involved, it would be difficult to control major outbreaks. Thus, understanding the cause and effect relationship between initial small outbreaks and large outbreaks that occur later in the year is crucial for the control of the pest.

Recently, molecular markers have been used to enhance understanding of insect displacements, especially including estimates of movement of particular genotypes and/ or biotypes, reproductive strategy and success. Such approaches have also been used to study founder events [5], geographical invasions [6], small and large scale displacements [7,8], including movement of entire population demes [9], and even altitudinal movements related to habitat patchiness and persistence [10]. Molecular data can yield valuable information when integrated with information from ethology, field ecology, comparative morphology, systematics and palaeontology [11]. Use of direct and indirect methods of tracking insects along with description of the role and utility of various molecular markers – protein and DNA – in monitoring insect dispersal, has been extensively reviewed [12].

Arbitrarily-primed DNA markers, and involving the polymerase chain reaction (PCR), have proved very useful for genetic fingerprinting and for facilitating positional cloning of genes. This class of markers are particularly important for less studied species, for which genome sequence information is generally not known. These technologies include randomly amplified polymorphic DNA (RAPDs) [13,14], DNA amplification fingerprinting (DAF)[15], and amplified fragment length polymorphisms (AFLPs) [16]. In this study, we used a variant of the RAPD approach involving various nuclear and mitochondrial gene specific primers to trace the origin of teak defoliator outbreaks. It is expected that the molecular data would provide the necessary information to elucidate the origin of the epidemic population. Such information should prove valuable in planning and implementing measures to control these pests. Therefore, the aim of the present study was to identify the relationship among the three apparent populations – endemic, epicenter and epidemic.

## Results

The nuclear and mitochondrial gene specific primers chosen did not produce any amplification product when used in combination with the corresponding primers as described in the UBC primer set kit [17]. This resulted in our devising a novel PCR, which we have named RAGEP-PCR. In RAGEP-PCR, we used single nuclear and mitochondrial gene encoding primers at low stringency annealing temperatures. Unlike RAPDs, in RAGEP longer nuclear (21–26 nucleotide) and mitochondrial (19–26 nucleotide) gene encoding primers were utilised, and which we have here extensively employed to evaluate the species taxonomic specificity/reproducibility and to discriminate the endemic, epicenter and epidemic populations of teak defoliator from one another.

**Figure 1:**
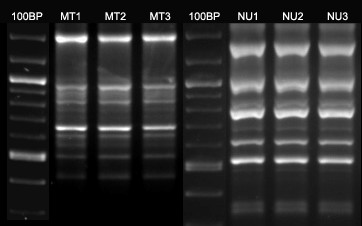
Reproducibility of RAGEP fingerprints. mt1-3, nu1-3 depicts reproducibility of RAGEPs using mitochondrial and nuclear primers respectively

**Figure 2:**
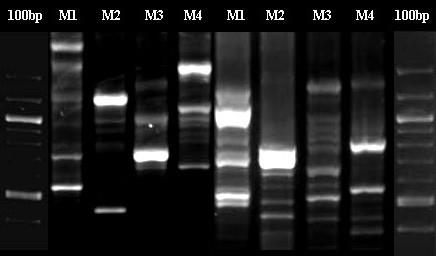
Species specificity of RAGEP fingerprints. M1-4 depicts variability in Lepidopteran species. *M1-Eutectona machaeralis*, *M2-Sylepta derogata*, *M3-Cnaphalocrocis medinalis*, *M4-Bombyx mori*

RAGEP markers were first tested for polymorphisms, species-specificity and repeatability. Similar fingerprinting pattern were observed in subsequent PCRs for the same individual using the same primers (Fig 1), which displayed overall robustness and repeatability with RAGEP-PCR. It was also possible to discriminate various moth species based on their species-specific DNA fingerprint pattern (Fig 2). The bands scored for each nuclear RAGEP used in the present study were of a size range 200 bp to 1500 bp. With nuclear RAGEP markers, an average of 2–3 monomorphic bands were observed, except for primer CK6-5'. In each marker, the average number of bands scored varied from 7–16. The maximum number of bands was detected using primer cytC-B-3', while the maximum number of monomorphic bands were detected using primer EFS599.

**Figure 3:**
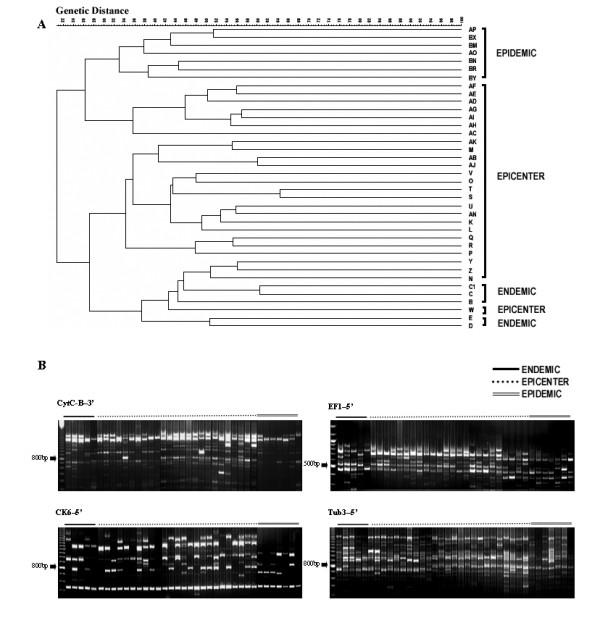
RAGEP fingerprint patterns generated by nuclear gene specific DNA markers in individuals of three populations (Panel B) and UPGMA dendrogram showing clustering of different insect populations of *Hyblaea puera* (Panel A)

Each individual RAGEP marker gel was screened and a similarity matrix was generated. Subsequently similarity matrixes of all experimental patterns were combined to generate a UPGMA (Unweighted pair-group mathematical average) tree. While evaluating the similarity matrix based on the Dice coefficient for all nuclear specific RAGEP markers and whilst constructing a UPGMA tree, it was observed that the various population groups of *H. puera *fall in two clusters, which are further divided into two major sub clusters. Average similarity between the two major clusters was 20%, while that between the two sub clusters was 34%. In one of the major clusters, we observed all the endemic insects clustering together with some of the populations from the epicenter insects; however, both populations fall in two distinct sub-clusters (Fig 3). Similarly in the second major cluster, the remaining populations from the epicenter and entire epidemic insect populations were likewise seen to fall into two distinct sub-clusters.

Using the mitochondrial RAGEP markers, the average numbers of bands scored for each primer ranged from 6–15. All bands scored were of size range 300 bp to 1600 kb. The maximum numbers of bands detected was found using primer SR-J-14233, the minimum numbers using marker N4-N-8924. Among mitochondrial markers, an average of 1–2 monomorphic bands were observed. The maximum number of monomorphic bands was observed using marker CB-N-10920.

**Figure 4:**
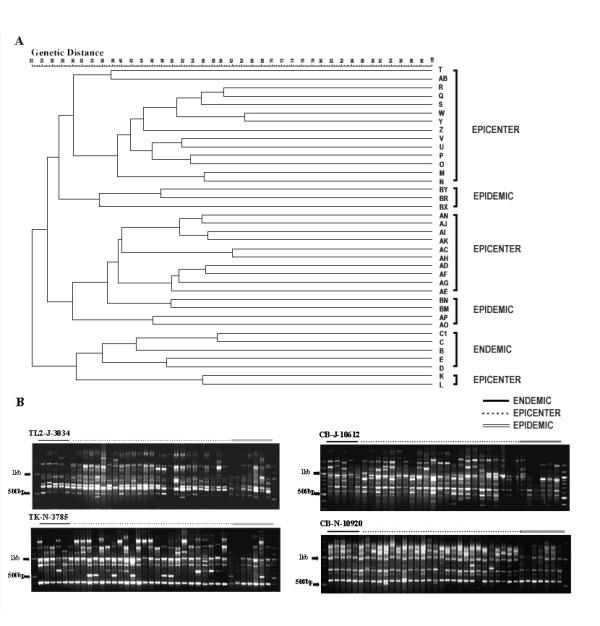
RAGEP fingerprint patterns generated by mitochondrial gene specific DNA markers in individuals of three populations (Panel B) and UPGMA dendrogram showing clustering of different insect populations of *Hyblaea puera* (Panel A)

Two distinct clusters were observed in the UPGMA dendrogram for mitochondrial markers. Similarity between the two clusters was only 20%. One of these clusters comprised the majority of the endemic samples with a few samples from epicenter insects, whilst the other cluster was comparatively larger and had the two major sub clusters. Both these sub-clusters have insects from epicenter and epidemic populations (Fig 4). From this dendrogram, it may be deduced that all the seven epidemic population samples tested in the study shared the same gene pool with sets of epicenter populations. In contrast, the endemic populations are genetically distant from the epicenter populations.

## Discussion

The first teak plantation in India was started as early as 1842 in Nilambur, Kerala State, India. Preliminary information on the life history of *H. puera *and the nature of its damage was published in 1898 [18]. *H. puera *outbreaks have been reported to begin in small epicenters and later spread to larger areas. It was then suspected that population build-up in the early outbreak epicenters might account for the subsequent widespread epidemic. However, a study using the time lapse (developmental time) between two epidemics to determine whether an earlier epidemic was responsible for causing the subsequent outbreak showed that all subsequent outbreaks could not be attributed to previous outbreaks, thereby indicating the possibility of migrant populations being involved [19].

Several technical advancements on the DNA fingerprinting methodologies have been established to resolve the taxonomic uncertainties and address the issue on species variability and migration [13-16,20,21]. The RAGEP-PCR method described here uses gene-specific primers and randomly amplifies the nuclear and mitochondrial-like gene products. Longer mitochondrial (19–26 nucleotide) gene encoding primers are likely to increase the reproducibility and specificity when compared to RAPD technique. This method was found to be efficient, simple and highly reproducible. Here it has been effectively used to discriminate the various population groups of *H. puera *infesting teak plantations in South India. It can also be used to discriminate taxonomically various closely – related moths to the species level. Mitochondrial RAGEP fingerprints are derived from the randomness of RAGEP-PCR. It is difficult to predict with certainty that the bands are diagnostic feature of the mitochondrial genome, but since RAGEP PCR uses gene specific primers, the PCR products could therefore be a result of amplification of homologous genes or pseudogenes which could represent nuclear mitochondrial DNA (NUMTs). Mitochondrial DNA sequences are frequently transferred to the nucleus-giving rise to NUMTs, which are considered to be common in eukaryotes [22]. Very high rate of horizontal transfer between organellar and nuclear genomes has been reported in the brown mountain grasshopper, *Podisma pedestris *(L.)[23].

Age groups, sexes, life history variants, etc. and the processes including birth, death, immigration and emigration as different phenotypic classes have been very well defined [24]. While studying the differentiation process of grain aphid, *Sitobion avenae *(F.) populations across agricultural ecosystems using DNA fingerprinting [(GATA)_4_] and RAPDs, it was possible to discriminate the micro- and macro geographical heterogeneity [25]. Highly diagnostic banding patterns in individuals of *S. avenae *on wheat and cocksfoot grass, *Dactylis glomerata *(L.) were observed during the early months of infestation, which declined as the season progressed, largely as a result of genetic drift and local movement between adjacent host species [26]. Monophyly and a strong biogeographic pattern of each biotype have been reported in whitefly, *Bemisia tabaci *(Gennadius) populations studied throughout the world [27]. While evaluating the genetic structure in introduced population of the fire ant, *Solenopsis invicta *(Buren) using different classes of markers, it was confirmed that both mitochondrial and nuclear markers display the same hierarchical structure [28]. Distinct mitochondrial and nuclear DNA sequence divergence patterns for phylogenetic inference has been established among nymphalid butterflies [29].

The present study using RAGEP-PCR provides a tool for a logical continuation of the earlier work to trace the relationship of endemic, epicenter and epidemic populations of the teak defoliator. The dendrogram produced from nuclear RAGEP clearly indicates that the endemic insects are not involved in causing the epidemic; however, they are apparently involved in the localized spread by building up small epicenter populations. Similarly, while evaluating the observation based on mitochondrial RAGEP's, it is further apparent that endemic populations were not involved in causing the epidemic. This suggests that all the epidemic insects, which are spatially distinct, but temporally co-occurring, share the same gene pool.

Randomness of genome amplification methods have been efficiently used in constructing the phylogenetic history in the weevil, *Aubeonymus mariafranciscae *(Roudier), which had diverged recently [5], whilst the origin of the Argentine stem weevil, *Listronotus bonariensis *(Kuschel) in New Zealand, was traced to the eastern coast of South America [30]. Use of RAPDs to examine, for example, population subdivision of the saw toothed grain beetle, *Oryzaephilus surinamensis *(L.) [31], characterization and identification of Asian and North American gypsy moth, *Lymantria dispar *(L.) [32], host based genotype variation in *S. avenae *[33], and genotypic variation among different phenotypes of asexual adult winged and wingless of some clones of cereal aphid species [34], has been well documented. Earlier reports involving molecular DNA markers mention the use of these markers in the detection of sibling species of black flies, *Simulium *spp. [35], whilst the dynamics of colonization of *Drosophila subobscura *(Collin) [36] in the west coast of North America and its impact in the sibling species *Drosophila athabasca *Sturtevant and Dobzhansky, and *Drosophila azteca *Sturtevant and Dobzhansky has been extensively studied by allozymes, mitochondrial DNA (mtDNA) and RAPD markers.

With the Teak defoliator, earlier studies based on temporal and spatial distribution of the larvae indicated that the epicenters were not constant over the years and did not represent highly favourable local environments [3]. The present study found little evidence to show that the aggregation of moths belonging to the endemic populations cause the epicenter populations. On the other hand, the findings do suggest the alternate hypothesis, i.e., that immigration of moths from distant teak plantations cause the epidemic, and that there is a continuous inflow of moths during the infestation period. This suggests that under a single demographic structure, two phenotypic classes of *H. puera *coexist during the outbreak season. The degree of variability observed for RAGEPs also argues that this technique could be useful for a variety of questions, including individual identification, strain identification and phylogenetic analysis.

## Conclusions

The present results appear to validate the hypothesis, that control of *H. puera *epicenter populations would help prevent large-scale outbreaks of the teak defoliator in teak plantations. Therefore, appropriate strategies should be adopted to control the epicenter populations, which occurs in a smaller area. This appears to be a more practical and economical approach for teak defoliator management when compared with management of the pest in the total plantation area covering thousands of hectares. Thus the molecular markers detected using RAGEP-PCR can enhance the understanding of insect population dynamics and aid in tracing the spread and cause of epidemics.

## Methods

### Sample collection

**Figure 5:**
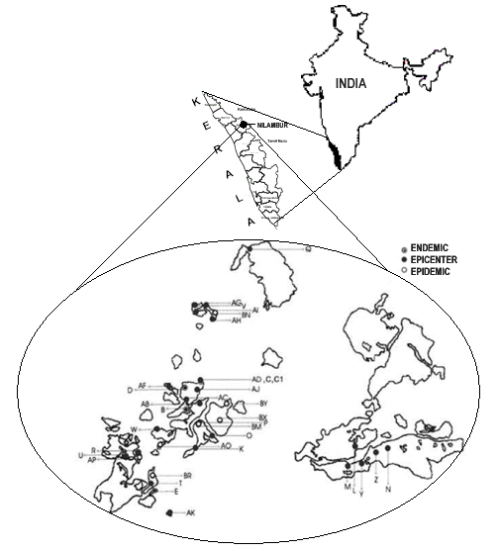
Landscape of Nilambur teak plantation showing distribution of the endemic, epicenter and epidemic populations of *Hyblaea puera*

Based on the spatial pattern of infestation in the past, the area was divided into convenient observation units of approximately 50 ha, based on natural boundaries of streams, roads and footpaths. The canopy of teak is continuous within in the observation area. Each area was monitored every 15 days, which was precisely based on the life cycle of *H. puera*. Larval samples were collected from the infestation sites. Whenever fifth instar larvae were available, ten larvae were preserved in 70 % alcohol and stored deep frozen at -20°C. If only lower stages were available, i.e., third or fourth instar, they were reared up to 5th instar in the laboratory. Ten 5th instar larvae were preserved for DNA isolation from each sample site, whilst the remaining larvae were reared into the next generation. Each sample was assigned a code number containing the details of Year / Month / Date / Block / Grid / Generation for further reference. Using the duration of each instar (egg – one day; 1^st ^and 2^nd ^instars – two days each, 3^rd ^to 5^th ^instars – three days each; pre-pupa – one day and pupa – four days), the temporal data on outbreaks were examined to see whether each subsequent epidemic could be explained on the basis of a previous outbreak. The details of location of pest incidence and extent of infestation were later transferred to the field map in order to understand the spatial pattern of infestation (Fig 5).

Populations were classified as 'endemic', 'epicenter' and 'epidemic', based on their time of occurrence and the density of the population as represented by the area it infests. Five endemic populations, twenty six epicenter populations and seven epidemic populations for the year 2002 were included in the study. Earlier studies had indicated that outbreak begin as small epicenters in February during the pre-monsoon season and end by June. Endemic samples were collected throughout that year based on their stray occurrences in various life stages, whilst epicenter samples from each aggregated patch were collected only from the insects that attained the same stage of its life cycle at the time of collection in that patch. Similarly the epidemic samples were also collected from insects representing the same life stages at the time of collection from each aggressive patch. The temporal relationship between the endemic population and the epicenter populations and that of the epicenter populations with the large-scale epidemics were first worked out. The larval samples that were geographically close and had a difference of one complete life cycle stage between the population groups were subjected to molecular studies to evaluate their relatedness.

### DNA isolation

DNA extraction was performed with a minor modification of isolation and purification protocol as described earlier [37] being extracted from whole larvae and quantified spectrophotometrically using a spectrophotometer at 260 nm (Shimadzu). The quality of the DNA was checked spectrophotometrically by taking the absorbance ratios of 260/280 nm.

### Polymerase chain reaction

Both nuclear and mitochondrial DNA RAGEP amplifications were performed in a total volume of 30 μl. Each reaction consisted of 1x Taq buffer with 1.5mM MgCl_2_, 1.2 U of *Taq *polymerase (BG), 0.25 mM of dNTPs (Amersham) and 12 pM of primer per reaction. Primers were initially screened for polymorphism and repeatability. Amplifications were performed in similar cycling conditions in a Thermocycler (Biorad) programmed as follows: initial denaturation at 95°C for 5 min., followed by 45 cycles of cycle denaturation at 94°C for 1 min., annealing at 36°C for 1 min., extension at 72°C for 2 min. and final extension at 72°C for 5 mins. The amplification products were separated using 1.2% agarose gel in 0.5 × TBE buffer with ethidium bromide staining to visualize the product separation using a Bio-Rad's Fluor S imager. The molecular weight of each band was estimated by comparing with a co-migrating 100-bp ladder (Amersham). RAGEP fingerprints of each sample from different regions were then interpreted using Fingerprint type module of Bionumerics software (Applied Maths Kortrijk Belgium, ver.2.0).

A preliminary screening with 50 nuclear RAGEPs and 37 mitochondrial RAGEP primers were evaluated for polymorphism and repeatability. Only 11 nuclear (Table-1) and mitochondrial RAGEPs (Table-2) from each group was selected for the study, as they showed constant repeatability of highly polymorphic patterns. Species specificity was evaluated by comparing the banding patterns in *H. puera *with those from the Teak skeletonizer, *E. machaeralis *(Walk.), Leaf roller, *Sylepta derogata *(F.), Leaf folder, *Cnaphalocrocis medinalis *(Guenée), and the Silkworm, *Bombyx mori *(L.).

**Table 1:** Insect nuclear gene specific primer sequences used in nuclear RAGEP-PCR

S.No	Primer name	Sequence
1	EFS599	ATC TCC GGA TGG CAC GG(CT) GAC AA
2	22.5drc	GAA CCA (AG)TT (AG)AC (AG)TG (AG)AA GAT C
3	LEPWG1	GA(AG)TG(CT)AA(AG)TG(CT)CA(CT)GG(CT)ATGTCT GG
4	CK6-5'	GAC CAC CTC CGA GTC ATC TC(CG) ATG
5	CK7-3'	CAG GTG CTC GTT CCA CAT GAA
6	CytC-B-3'	CAT CTT GGT GCC GGG GAT GTA TTT CTT
7	EF1-5'	GAC AAC GTT GGC TTC AAC GTG AAG AAC G
8	Tub3-5'	GAT TTG GAG CC(AGCT) GG(AGCT) ACC ATG GA
9	18S-A1984	TCC CTG GTT GAT CCT GCC AGT A
10	S1124	AGC GTA TGG C(AC)T C(AG)A AGAACT G
11	rcM4	ACA GC(CGA) AC(GT) GT(TC) TG(CT) CTC AT(AG) TC

**Table 2:** Insect mitochondrial gene specific primer sequences used in mitochondrial RAGEP-PCR

S.No	Primer name	Sequence
1	C1-J-2183	CAA CAT TTA TTT TGA TTT TTT GG
2	TL2-J-3034	AAT ATG GCA GAT TAG TGC A
3	C2-N-3661	CCA CAA ATT TCT GAA CAT TGA CCA
4	TK-N-3785	GTT TAA GAG ACC AGT ACT TG
5	N4-N-8924	AAA GCT CAT GTT GAA GCT CC
6	CB-J-10612	CCA TCC AAC ATC TCA GCA TGA TGA AA
7	LR-J-12887	CCG GTC TGA ACT CAG ATC ACG T
8	LR-J-13417	ATG TTT TTG TTA AAC AGG CG
9	LR-N-13398	CGC CTG TTT AAC AAA AAC AT
10	SR-J-14233	AAG AGC GAC GGG CGA TGT GT
11	CB-N-10920	CCC TCA GAA TGA TAT TTG TCC TCA

### Analysis

The polymorphic content for nuclear and mitochondrial primers were analyzed using Bionumerics software [38]. Band search parameters were kept constant as 5% minimum profiling for all the gels. The position tolerance for selection of bands in constructing a dendrogram was kept constant at 1% through out the interpretations. Only bands showing clear and reproducible patterns were included in the final analysis and these were scored. Real-time normalization of gel electrophoresis patterns and band position for all the gels was based on the reference system for the species-specific bands. Normalization helped us to control the brightness and streakiness of bands without altering the lighter bands and also control the inter-gel mobility shifts. Subsequently a data matrix of similarity values was produced for each individual for each marker. The Dice coefficient was used to analyze the similarities of the banding patterns. Consensus similarity matrix and dendrogram based upon individual matrices from different markers were used for pair wise clustering based on unweighted pair group method (UPGMA) with average linkages (11). The UPGMA dendrogram prevails on the assumption that nucleotide substitution rates are same across all branches. It employs a sequential clustering algorithm, in which local topological relationships are identified in order of similarity, and the phylogenetic tree is built in a stepwise manner. All analysis was done using Bionumerics software V-2.

## Authors' contributions

CN and MB performed the molecular studies and are responsible for the interpretation of molecular data whilst TV and VVS performed the field data collection and are responsible for spatial and temporal data interpretation. All authors read and approved the final manuscript. MB and VVS conceived and designed the study.

## References

[B1] Browne FG (1968). Pests and Diseases of Forest Plantation Trees.

[B2] Nair KSS, Sudheendrakumar VV, Varma RV, Chacko KC (1985). Studies on the seasonal incidence of defoliators and the effect of defoliation on volume increment of teak. Kerala Forest Research Institute, Peechi, Kerala. KFRI Research Report No 30.

[B3] Nair KSS, Sudheendrakumar VV (1986). The teak defoliator, *Hyblaea puera *: Defoliation dynamics and evidence for short-range migration of moths. Proc Indian Natl Sci Acad Part B Biol Sci.

[B4] Nair KSS, Mohanadas K (1996). Early events in the outbreak of teak caterpillar *Hyblaea puera*. Intl J Ecol Environ Sci.

[B5] Taberner A, Dopazo J, Castanera P (1997). Genetic characterization of populations of a de novo arisen sugar beet pest, *Aubeonymus mariaefranciscae *(Coleoptera, Curculionidae), by RAPD analysis. J Mol Evol.

[B6] Stone GN, Sunnucks P (1993). Genetic consequences of an invasion through a patchy environment – the cynipid gall wasp *Andricus quercuscalicis *(Hymenoptera: Cynipidae). Mol Ecol.

[B7] Loxdale HD, Lushai G, Woiwod IP, Reynolds DR, Thomas CD (2001). Use of genetic diversity in movement studies of flying insects. Insect Movement: Mechanisms and Consequences.

[B8] Daly JC, Gregg P (1985). Genetic variation in Heliothis in Australia: species identification and gene flow in two pest species *H. armigera *(Hubner) and *H. punctigera Wallengren *(Lepidoptera: Noctuidae). Bull Entomol Res.

[B9] Loxdale HD, Lushai G (1999). Slaves of the environment: the movement of insects in relation to their ecology and genotype. Phil Trans R Soc Ser B.

[B10] Liebherr JK (1988). Gene flow in ground beetles (Coleoptera:Carabidae) of differing habitat preference and flight-wing development. Evolution.

[B11] Avise JC (1994). Molecular markers, natural history and evolution.

[B12] Osborne JL, Loxdale HD, Woiwod IP, Bullock JM, Kenward RE, Hails RS (2002). Monitoring insect dispersal: methods and approaches. Dispersal ecology pp24–49, British ecological Symposium, 3–5, 2001.

[B13] Welsh J, McClelland M (1990). Fingerprinting genomes using PCR with arbitrary primers. Nucleic Acids Res.

[B14] Williams JGK, Kubelik AR, Livak KJ, Rafalski JA, Tingey SV (1990). DNA polymorphisms amplified by arbitrary primers are useful as genetic markers. Nucleic Acids Res.

[B15] Caetano-Anolles G, Bassam BJ, Gresshoff PM (1991). DNA amplification fingerprinting using very short arbitrary oligonucleotide primers. Biotechnology.

[B16] Vos P, Hogers R, Bleeker M, Reijans M, van de Lee T, Hornes M, Frijters A, Pot J, Peleman J, Kuiper M (1995). AFLP: a new technique for DNA fingerprinting. Nucleic Acids Res.

[B17] University of British Columbia. http://www.michaelsmith.ubc.ca/services/NAPS/.

[B18] Bourdillon TF (1898). Insect attacking teak in southern India. Indian Forester.

[B19] Nair KSS, Sudheendrakumar VV, Varma RV, Mohanadas K (1998). Tracing the epicenters of teak defoliator outbreaks in Kerala. Kerala Forest Research Institute, Peechi, Kerala. KFRI Research Report No 147.

[B20] Schlipalius DI, Waldron J, Carroll BJ, Collins PJ, Ebert PR (2001). A DNA fingerprinting procedure for ultra high-throughput genetic analysis of insects. Insect Mol Biol.

[B21] Hebert PD, Ratnasingham S, deWaard JR (2003). Bar coding animal life: cytochrome c oxidase subunit 1 divergences among closely related species. Proc R Soc Lond B Biol Sci.

[B22] Richly E, Leister D (2004). NUMTs in sequenced eukaryotic genomes. Mol Biol Evol.

[B23] Bensasson D, Zhang DX, Hewitt GM (2000). Frequent assimilation of mitochondrial DNA by grasshopper nuclear genomes. Mol Biol Evol.

[B24] Roderick GK (1996). Geographic structure of insect populations: Gene flow, phylogeography and their uses. Ann Rev Entomol.

[B25] De Barro PJ, Sherratt TN, Wratten S, Maclean N (1994). DNA fingerprinting of cereal aphids using (GATA)4. Eur J of Entomology.

[B26] De Barro PJ, Sherratt TN, Brookes CP, David O, Maclean N (1995). Spatial and temporal variation in British field populations of the grain aphid *Sitobion avenae *(F) (Hemiptera: Aphidae) studied using RAPD -PCR. Proc Of R Soc Lond B.

[B27] De Barro PJ, Driver F, Trueman JW, Curran J (2000). Phylogenetic relationships of world populations of *Bemisia tabaci *(Gennadius) using ribosomal ITS1. Mol Phylogenet Evol.

[B28] Ross KG, Shoemaker DD, Krieger MJ, DeHeer CJ, Keller L (1999). Assessing genetic structure with multiple classes of molecular markers: a case tudy involving the introduced fire ant *Solenopsis invicta*. Mol Biol Evol.

[B29] Brower AV, DeSalle R (1998). Patterns of mitochondrial versus nuclear DNA sequence divergence among nymphalid butterflies: the utility of wingless as a source of characters for phylogenetic inference. Insect Mol Biol.

[B30] Williams CL, Goldson SL, Baird DB, Bullock DW (1994). Geographical origin of an introduced insect pest, *Listronotus bonariensis *(Kuschel), determined by RAPD analysis. Heredity.

[B31] Brown RJ, Malcolm CA, Mason PL, Nichols RA (1997). Genetic differentiation between and within strains of the saw-toothed grain beetle, *Oryzaephilus surinamensis *(Coleoptera: Silvanidae) at RAPD loci. Insect Mol Biol.

[B32] Garner KJ, Slavicek JM (1996). Identification and characterization of a RAPD-PCR marker for distinguishing Asian and North American gypsy moths. Insect Mol Biol.

[B33] Lushai G, Markovitch O, Loxdale HD (2002). Host-based genotype variation in insects revisited. Bull Entomol Res.

[B34] Lushai G, Loxdale HD, Brookes CP, von Mende N, Harrington R, Hardie J (1997). Genotypic variation among different phenotypes within aphid clones. Proc R Soc Lond B Biol Sci.

[B35] Brockhouse CL, Vajime CG, Marin R, Tanguay RM (1993). Molecular identification of onchocerciasis vector sibling species in black flies (Diptera: Simuliidae). Biochem Biophys Res Commun.

[B36] Pascual M, Balanya J, Latorre A, Serra L (1997). Diagnosis of sibling species of Drosophila involved in the colonization of North America by *D. subobscura*. Mol Ecol.

[B37] Andrew FC, Gary AF, Clapp JP (1995). Isolation and purification of Insect DNA. Methods in Molecular Biology.

[B38] Bionumerics software. Applied Maths Kortrijk Belgium, ver20.

